# Brazilian spatial, demographic, and socioeconomic data from 1996 to 2020

**DOI:** 10.1186/s13104-022-06044-w

**Published:** 2022-05-10

**Authors:** Ronaldo Fernandes Santos Alves, Patricia de Moraes Mello Boccolini, Lais Ribeiro Baroni, Laís de Almeida Relvas-Brandt, Raquel de Abreu Junqueira Gritz, Cristiano Siqueira Boccolini

**Affiliations:** 1 Institute of Scientific and Technological Communication and Information in Health, Oswaldo Cruz Foundation, Fiocruz, Rio de Janeiro, Brazil; 2 Núcleo de Informação, Políticas Públicas e Inclusão Social, NIPPIS, UNIFASE, Petrópolis, Brazil; 3 Federal Center for Technological Education of Rio de Janeiro, CEFET/RJ, Rio de Janeiro, Brazil

**Keywords:** Brazil, Population characteristics, Socioeconomic factors, Vital statistics, Routinely collected health data, Health information systems, Database, Metadata

## Abstract

**Objectives:**

We present a database on Brazilian spatial, demographic, and socioeconomic characteristics from 1996 to 2020. This database aims for integration and harmonization with epidemiological data from two major studies. It can also be a valuable database for designing and conducting various types of epidemiologic research, such as health inequality studies, ecological studies (mapping and time-trends), and multi-level analysis.

**Data description:**

The database gathers official information obtained via open sources from the Brazilian Institute of Geography and Statistics, the Institute for Applied Economic Research, and the Ministry of Health. It includes 139,153 observations and 26 attributes aggregated by years and policy-relevant geographic units on geocoding of municipality centroids, total population size, child population by age-group, birth and mortality measures, Brazilian Municipal Human Development Index, Gini coefficient, Gross Domestic Product, and sanitation. We automated all data processing and curation in the free and open software R.

## Objective

Spatial, demographic, and socioeconomic information is crucial for research, planning, and policy development in health and other sectors. It helps countries compute many health indicators, optimize budgeting and resources allocation, measure and track progress toward international goals and national priorities, and support effective decision-making [1, 2]. Brazil is a federative republic with more than 211 million inhabitants, representing 47% of South America total population, and a well-developed national statistical system with 98% of births and 96% of deaths registered [3].

We present a database on Brazilian spatial, demographic, and socioeconomic characteristics from 1996 to 2020. This database aims for integration and harmonization with epidemiological data from two major studies [4, 5], including avoidable child mortality, hospitalization, immunization, breastfeeding, and primary health care resources [6]. It can also be a valuable database for designing and conducting various types of epidemiologic research, such as health inequality studies, ecological studies (mapping and time-trends), and multi-level analysis.

## Data description

The database gathers official information obtained via open sources from the Brazilian Institute of Geography and Statistics (IBGE) [7, 8], the Institute for Applied Economic Research (IPEA) [9], and the Ministry of Health (MoH) [10, 11]. Data extraction occurred on November 18, 2021. The database has 139,153 observations and 26 attributes aggregated by years (1996–2020) and policy-relevant geographic units (country, macroregions, states, municipalities, and capitals). It includes geocoding of municipality centroids, total population size, child population by age-group, birth and mortality measures, Brazilian Municipal Human Development Index (MHDI), Gini coefficient, Gross Domestic Product (GDP), and sanitation. We automated all data processing and curation in the free and open software R. The codes can be audited, replicated, and reused to produce alternative analysis.

Table 1 provides an overview of the report’s files and datasets stored in Synapse. The R scripts hold the codes for the data extraction (data files 1–5), transformation, and loading (data files 6–11) routines. We extracted the data in its original format (datasets 1–5) and separately saved each workflow endpoint’s processed data (datasets 6–11). The HTML files show type-specific information for all attributes of the treated datasets, including statistical summaries and missing frequencies (data files 12–17). Data file 11 builds the database (dataset 11), and data file 18 documents its metadata and attribute descriptions.

**Table 1:** Overview of data files/data sets

Label	Name of datafile/datasets	File type (file extension)	Data repository and identifier
Data file 1	script_ibge_webscraping	R code (.r)	Synapse: 10.7303/syn26525521 [12]
Data file 2	script_moh_webscraping	R code (.r)	Synapse: 10.7303/syn26525521 [12]
Data file 3	script_ipea_webscraping	R code (.r)	Synapse: 10.7303/syn26525521 [12]
Data file 4	script_sinasc_webscraping	R code (.r)	Synapse: 10.7303/syn26525521 [12]
Data file 5	script_sim_webscraping	R code (.r)	Synapse: 10.7303/syn26525521 [12]
Data file 6	script_ibge_ingestion	R code (.r)	Synapse: 10.7303/syn26525521 [12]
Data file 7	script_moh_ingestion	R code (.r)	Synapse: 10.7303/syn26525521 [12]
Data file 8	script_ipea_ingestion	R code (.r)	Synapse: https://doi.org/10.7303/syn26525521 [12]
Data file 9	script_sinasc_ingestion	R code (.r)	Synapse: 10.7303/syn26525521 [12]
Data file 10	script_sim_ingestion	R code (.r)	Synapse: 10.7303/syn26525521 [12]
Data file 11	script_master_basics	R code (.r)	Synapse: 10.7303/syn26525521 [12]
Data file 12	ibge_sprint_dataselfie	HTML (.html)	Synapse: 10.7303/syn26525521 [12]
Data file 13	moh_sprint_dataselfie	HTML (.html)	Synapse: 10.7303/syn26525521 [12]
Data file 14	ipea_sprint_dataselfie	HTML (.html)	Synapse: 10.7303/syn26525521 [12]
Data file 15	sinasc_sprint_dataselfie	HTML (.html)	Synapse: 10.7303/syn26525521 [12]
Data file 16	sim_sprint_dataselfie	HTML (.html)	Synapse: 10.7303/syn26525521 [12]
Data file 17	basics_master_dataselfie	HTML (.html)	Synapse: 10.7303/syn26525521 [12]
Data file 18	basics_master_overview	excel (.xlsx)	Synapse: 10.7303/syn26525521 [12]
Dataset 1	ibge_data_raw	zipped (.zip)	Synapse: 10.7303/syn26525521 [12]
Dataset 2	moh_data_raw	zipped (.zip)	Synapse: 10.7303/syn26525521 [12]
Dataset 3	ipea_data_raw	zipped (.zip)	Synapse: 10.7303/syn26525521 [12]
Dataset 4	sinasc_data_raw	zipped (.zip)	Synapse: 10.7303/syn26525521 [12]
Dataset 5	sim_data_raw	zipped (.zip)	Synapse: 10.7303/syn26525521 [12]
Dataset 6	ibge_clean_data	R data (.rdata)	Synapse: 10.7303/syn26525521 [12]
Dataset 7	moh_clean_data	R data (.rdata)	Synapse: 10.7303/syn26525521 [12]
Dataset 8	ipea_clean_data	R data (.rdata)	Synapse: 10.7303/syn26525521 [12]
Dataset 9	sinasc_clean_data	R data (.rdata)	Synapse: 10.7303/syn26525521 [12]
Dataset 10	sim_clean_data	R data (.rdata)	Synapse: 10.7303/syn26525521 [12]
Dataset 11	basics_master_data	R data (.rdata)	Synapse: 10.7303/syn26525521 [12]

Anyone can browse the content on the Synapse website, but you must register for an account using your email address to download the files and datasets

### Data construction

The data workflow comprises two main steps. The first step covered the extraction, transformation, and loading routines of data obtained from primary sources of information. The data extraction resulted in 1452 raw files, including spatial data of the Brazilian municipalities, individual data on births and deaths, and aggregated data on population size and socioeconomic characteristics. The key features of data transformation were (i) variables selection/renaming and observations filtering, (ii) calculation of municipality centroids, (iii) correction of codes and names identifying geographic units, (iv) cleansing numeric values, e.g., excluding special characters, and (v) enrichment of the municipal datasets with data aggregated by states, macroregions, and country. This step produced five datasets treated and usable in the database construction.

The second step in the workflow involved data integration, harmonization, and enrichment. The IBGE treated-dataset defined the final database structure, in which we combined the other treated datasets according to the years and codes of geographic units. As socioeconomic data was not available for all time points, we applied a simple imputation method for missing data using the next or previous observation of the geographic units. Furthermore, we created the following variables: mortality rate, infant mortality rate, birth rate, estimated population of children under 1-year-old and 1-year-old. The number of children by age group considered two business rules. For children under 1-year-old, we used the MoH estimates in 1996–2005 and the number of live births in 2006–2020. For children of 1-year-old, we used the MoH estimates in 1996–2005 and our estimates in 2006–2020 (calculation method: the difference between live births and infant deaths occurred in the previous year). R codes and data processing/curation were peer-reviewed, and their results compared to the information presented on official sites.

## Limitations

We should mention the potential limitations and warnings of the database. First, our eight socioeconomic indicators have different timeframes because of their availability at the municipal level—GDP total and per capita from 1999 to 2018. MHDI (global, education, longevity, and income dimensions), Gini coefficient, and sanitation only 1991, 2000, 2010. It’s worth noting that Brazilian National Household Sample Survey provides some of these indicators for capitals, states, macroregions, and Brazil with a longer timeframe. Moreover, we adopted a simple imputation method for missing data, with several intrinsic limitations, and we presented GDP indicators in Brazilian reais and unadjusted for purchasing power parity. Second, total population size came from the results of demographic censuses (2000, 2010), inter-census counts (1996, 2007), and population estimates (other years), the only ways to capture these data at the municipal level. Our results for states, macroregions, and Brazil may diverge somewhat from population projections, which do not incorporate post-baseline territorial boundary updates. Finally, the Live Birth Information System (SINASC) and the Mortality Information System (SIM), used to collect live births and deaths data, have variable coverages over time and across geographic units—i.e., lower at the beginning of historical series and underserved areas. Nevertheless, overall SINASC and SIM coverages are high—98% and 96%, respectively [3].

## Data Availability

The data described in this Data note are freely and openly available on the Synapse repository at 10.7303/syn26525521. Anyone can browse the content on the Synapse website, but you must register for an account using your email address to download the files and datasets. Please see Table 1 and references [4, 5, 12] for details and links to the data.

## Abbreviations

GDP: Gross domestic product
IBGE: Brazilian institute of geography and statistics
IPEA: Institute for applied economic research
MHDI: Brazilian municipal human development index
MoH: Ministry of health
SINASC: Live birth information system
SIM: Mortality information system
